# A Strategy for Dimensionality Reduction and Data Analysis Applied to Microstructure–Property Relationships of Nanoporous Metals

**DOI:** 10.3390/ma14081822

**Published:** 2021-04-07

**Authors:** Norbert Huber

**Affiliations:** 1 Institute of Materials Mechanics, Helmholtz-Zentrum Hereon, Max-Planck-Str. 1, 21502 Geesthacht, Germany; norbert.huber@hereon.de; Tel.: +49-4152-87-2501; 2 Institute of Materials Physics and Technology, Hamburg University of Technology, Eißendorfer Str. 42, 21073 Hamburg, Germany

**Keywords:** nanoporous metals, open-pore foams, FE-beam model, data mining, mechanical properties, hardness, machine learning, principal component analysis, structure–property relationship, microcompression, nanoindentation

## Abstract

Nanoporous metals, with their complex microstructure, represent an ideal candidate for the development of methods that combine physics, data, and machine learning. The preparation of nanporous metals via dealloying allows for tuning of the microstructure and macroscopic mechanical properties within a large design space, dependent on the chosen dealloying conditions. Specifically, it is possible to define the solid fraction, ligament size, and connectivity density within a large range. These microstructural parameters have a large impact on the macroscopic mechanical behavior. This makes this class of materials an ideal science case for the development of strategies for dimensionality reduction, supporting the analysis and visualization of the underlying structure–property relationships. Efficient finite element beam modeling techniques were used to generate ~200 data sets for macroscopic compression and nanoindentation of open pore nanofoams. A strategy consisting of dimensional analysis, principal component analysis, and machine learning allowed for data mining of the microstructure–property relationships. It turned out that the scaling law of the work hardening rate has the same exponent as the Young’s modulus. Simple linear relationships are derived for the normalized work hardening rate and hardness. The hardness to yield stress ratio is not limited to 1, as commonly assumed for foams, but spreads over a large range of values from 0.5 to 3.

## 1. Introduction

Nanoporous gold (np-Au) made by dealloying can be produced as macroscopic objects that exhibit a bi-continuous network of nanoscale pores and solid “ligaments” connected in nodes. An overview of the fascinating morphologies and mechanical properties of this material is provided in [1,2,3]. The skeleton of the structure is formed by ligaments, which can be controlled in their average diameter by altering the dealloying conditions, thus allowing one to examine the impact of the ligament size on the macroscopic mechanical properties [4,5]. It has been recently demonstrated that the dealloying process can be applied sequentially and allows one to produce hierarchically organized nanoporous metals with superior macroscopic properties compared to similar materials with only one hierarchy level [6].

So far, even for one hierarchy level, no model exists that allows for the prediction of the macroscopic mechanical properties based on the parameters used in the sample preparation. Recently, the evolution of the ligament size and the network connectivity during thermal treatment was modeled with kinetic Monte Carlo simulations [7] for a large range of solid fractions, but the connection to the macroscopic mechanical properties is still missing. For a selected microstructure, this is realized by conventional meshing and finite element (FE) simulation, e.g., as shown in [8], but from this point, it is still a long way to go towards an all-inclusive process–microstructure–property model that handles all required steps along a fully automated work flow and at the required efficiency. An overview of the elements needed for such a work flow based on efficient simulation models, data mining, and AI is presented in [9].

A key element represents the relationship that efficiently translates a set of microstructural parameters and material properties of the solid phase into macroscopic properties. Together with the structural information from, e.g., high-resolution 3D tomography and image analysis [10,11], all relevant aspects are currently under development. As pointed out in [9], they altogether will allow for an efficient scan of large multidimensional parameter spaces of descriptors and reliably predict the macroscopic mechanical properties for any assumed constitutive law on the level of a single ligament. Moving from scarce data to rich data allows for data mining of the fundamental structure–property relationships. The objective is to derive robust approximations that generalize the available data and support our understanding of the underlying physics well beyond the application of machine learning as a black box method.

In this work, we concentrate on the relationship that allows predicting mechanical properties based on microstructural information or, formulated as an inverse problem, enables us to determine microstructural descriptors from macroscopic test data. Due to the complex morphology of this material, FE modeling of np-Au with all its structural complexity is highly challenging. Two general paths exist, which are summarized in [9]. One route uses random structures (spinodal decomposition, leveled waves); the second is based on unit cells (Gibson–Ashby, gyroid, diamond). The mechanical behavior of random structures is usually predicted with molecular dynamics (MD) simulations [12,13,14] or with continuum mechanics using FE-solid or voxel models [8,15,16]. In combination with plasticity, also the FE-models lead to large computing times and allow only for a very limited number of simulations. Furthermore, the limited model size makes it extremely difficult to simulate a nanoindentation test that averages over sufficient features, such that it can be analyzed like an experiment. One of the rare examples that goes in this direction is the work of Farkas et al. [14], which presents a MD simulation of nanoindentation in a single crystal with a relative density of 0.67 and ligament diameter of 2 nm.

As pointed out in [9], FE-beam models provide the efficiency and flexibility needed for the generation of larger data sets and, at the same time, allow for an independent variation of all structural and material parameters of interest. This modeling technique has been successfully applied in studying the mechanical behavior of foams [17,18,19,20,21,22] and nanporous metals [10,23,24,25,26,27]. Research in this field concentrated mainly on the anisotropy of the macroscopic elastic properties as well as aspects of the structure–property relationships for elastic–plastic macroscopic compression. Until recently, the quantitative correct prediction of materials with relative densities >10% was limited to cylindrical ligament shapes [26]. The nodal correction proposed by Odermatt et al. enables us to expand FE simulations towards variations of the ligament shape from concave to convex [27]. The advantage of this approach is that the computational efficiency of FE-beam models is maintained. This paves the way for handling hundreds of simulations with a predictive model that is at the same time large enough for the simulation of nanoindentation.

The scope of this work is to study the influence of microstructure and material parameters on the macroscopic response of a porous metal. We will investigate the macroscopic behavior under compression as well as nanoindentation. For scanning the multidimensional parameter space, a highly efficient simulation model is required. Furthermore, the model set-up should allow for the independent variation of all important structural inputs. To this end, we use a representative volume element (RVE) that approximates the complex morphology of an open pore material by a diamond structure [23,24]. Using this unit cell, it is possible to define the degree of randomization and connectivity of the structure [28]. Together with the material parameters defining the mechanical behavior of the solid phase, this generates a highly dimensional parameter space that is hard to scan in a dense manner by numerical simulations. The dimensionality of the problem and limited number of simulations makes analysis of the underlying structure–property relationships very challenging. If we limit the number of parameters to five (two material and three microstructural parameters) and the number of variations per parameter to three, a systematic variation with one parameter at a time would end up with 243 simulations, which is already at the limit of the computer’s capacity. Adding more parameters or increasing the number of increments is almost impossible. Therefore, this investigation requires a strategy that exploits all available methods that contribute to reduce the dimensionality of the problem.

In this sense, the present work also serves as a guide, demonstrating how such a problem can be tackled systematically by means of a dimensional analysis, inclusion of a priori knowledge about the physical problem at hand, data generation strategies, principal component analysis, machine learning, and visualization. Along this path, Section 2 describes the FE-beam models used for generation of the data for macroscopic compression and nanoindentation. Section 3 and Section 4 deal with dimensionality reduction of the macroscopic compression and nanoindentation problem, respectively, where both sections follow the same methodology. Finally, it is shown that for important dependencies, simple mathematical formulations can be derived that relate the major influences of microstructure and mechanical properties to the macroscopic response.

## 2. FE-Model and Data Generation

FE-beam modeling is used to predict the macroscopic response of nanoporous metals during macroscopic compression and nanoindentation. The generation of the representative volume element (RVE) is established in the literature and is described only briefly in Section 2.1. In contrast, the simulation of nanoindentation is novel. The incorporation of a conical indenter and strategies for achieving an efficient simulation model that copes with the nonlinearities arising from the contact problem is described in Section 2.2.

### 2.1. Macroscopic Compression

**Figure 1:**
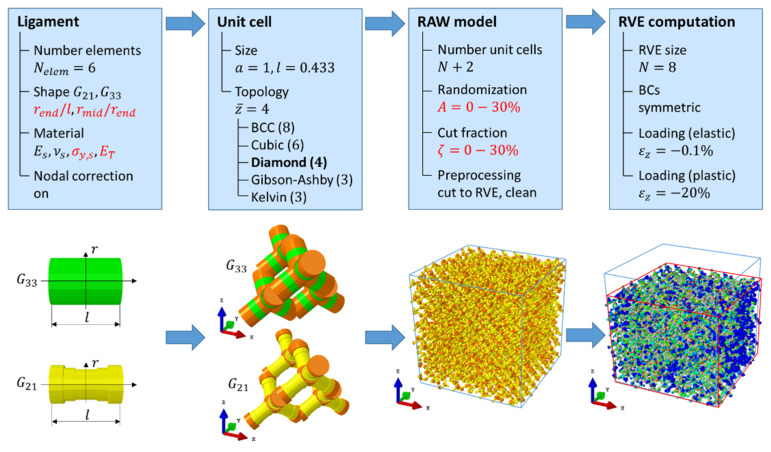
Workflow for generating a representative volume element (RVE) in four steps: (i) ligament, (ii) unit cell, (iii) RAW model after cutting and cleaning, and (iv) computation of the RVE. Varied parameters are highlighted in red color.

The FE software Abaqus was used for the numerical simulation of the RVE [29]. The model generation for macroscopic compression followed [23,24,27,28] and was organized hierarchically along the workflow presented in Figure 1. This workflow was programmed object oriented in Python with classes for the different hierarchy levels, allowing for scripting of the RVE generation and job submission within loops for the variation of input parameters. A postprocessing script handled the simulation analysis and database generation.

The model generation started at the ligament level, where the ligament axis is discretized in $N_{elem}$ FE beam elements with circular cross-section and variable radius r. The ligament shape is defined along the axis according to [10,11] by $r_{sym}^{*}=r_{mid}/r_{end}$ and $r_{end}/l$, where $r_{mid}$ and $r_{end}$ denote the ligament radius in the middle and at the ends, respectively, and l is the ligament length in a diamond unit cell. Together with the topology, the set of ligament geometry parameters $r_{mid}$, $r_{end}$, and l define the solid fraction $\varphi_{0}$ before randomization.

Odermatt et al. [27] developed nodal corrections for 16 ligament shapes that allow for a quantitative prediction of the elastic–plastic response of the RVE up to macroscopic strains of 20%. Details about the ligament geometries, initial solid fractions, and the nodal correction approach can be found in [27]. The extension of the nodal corrected zones is visible in the second column of Figure 1, where nodal corrected elements in the diamond unit cell are displayed in orange. With the nodal correction set “on”, their material parameters were modified such that the deformation behavior of the unit cell corresponded to that of an FE solid model of the same ligament shape. Preliminary simulations for decreasing number of elements using a unit cell with periodic boundary conditions confirmed that the nodal correction by [27] performed well in the range from 20 down to 6 FE elements per ligament for both geometries listed in Table 1. Therefore, 6 elements per ligament were chosen in this work for which the relative error in macroscopic stiffness and strength was within 15% error relative to the results of the FE solid model.

For generating an RVE of size  N, the unit cell is copied N+2 times (origin at [−1,−1,−1]) in each coordinate direction, followed by the randomization of the structure. The degree of randomization is defined by the parameter A, which corresponds to a random displacement in space applied to the connecting nodes by an amplitude A, which is given as a fraction of the unit cell size a [23,24]. Alternatively, one can also choose to displace an FE node in the mid-section of the ligaments by this magnitude normal to the ligament axis [27]. The randomization can be calibrated via the elastic Poisson’s ratio and is typically A=0.23 [24].

Because the coordination of the diamond structure of $\bar{z}=4$ is too high in comparison to experimental observations [10,30,31], the connectivity can be reduced by random cutting of a fraction ζ of the ligaments [28]. For diamond, the percolation threshold is reached for sufficiently large RVEs at a cut fraction of ζ→0.5, where the average coordination number approaches $\bar{z}\to 2$. For models of smaller size the percolation threshold is reached at lower values and is sensitive to the random realization. In combination with randomly cut ligaments, the randomization A can be reduced to values close to 0 to reach the elastic Poisson’s ratio measured in experiments [28]. Therefore, we chose these two parameters independent of each other and within comparably large ranges of 0≤A≤0.3 and 0≤ζ≤0.3.

The resulting RAW model of size 10×10×10 unit cells is randomly distorted and can contain free floating ligaments due to random cuts. A cleaned RVE is generated by cutting the RAW model to a cubic volume of size N=8 (origin at [0,0,0]) by removing all elements outside of this volume. Free floating ligaments are removed by two subsequent cleaning cycles that eliminate dangling ligaments and then re-attach element by element those ligaments that are connected to the residual core of the ligament network. For more details, the reader is referred to the supplementary material that is provided in [28]. The result of the preprocessing is an RVE of dimensions 8×8×8 unit cells with plane boundaries, consisting of 512 diamond unit cells with a total of 8192 ligaments and 49,152 FE-elements (A=0, ζ=0). Symmetry boundary conditions are applied to FE-nodes in the planes x=0, y=0, and z=0, while macroscopic compression is applied at the top face at the position z=N.

For simplicity, the model was generated such that the unit cell size corresponds to a unit size of 1 mm. Realistic microstructural dimensions of the ligament and the pore size can be achieved by self-similar scaling of the model to a desired characteristic size, e.g., a ligament diameter of 20–150 nm [5]. Because the material law does not account for size effects, the resulting macroscopic behavior is not affected by such a scaling. However, when the effect of the surface energy is included, the ligament size is important; then also the applied electrode potential must be defined [32]. These two parameters allow for switching of the strength and the plastic Poisson’s ratio during macroscopic deformation of the material.

A and ζ are dimensionless structural parameters describing the random distortion of the connecting nodes as fraction of the unit cell size and the fraction of randomly cut ligaments, respectively. Both parameters modify the solid fraction relative to the initial solid fraction $\varphi_{0}$. According to Roschning et al. [24], we should account for the distortion of the ligament axis by A by an increase in solid fraction by using

(1) $$\frac{\varphi_{A}}{\varphi_{0}}=1+0.15A+2.91A^{2},$$

whereas the random cutting ζ removes a fraction of ligaments and, therefore, mass from the model [28]

(2) $$\frac{\varphi_{\zeta }}{\varphi_{0}}=1-\zeta .$$

If the RVE is large enough, Equations (1) and (2) can be combined as

(3) $$\frac{\varphi }{\varphi_{0}}=\left(1-\zeta \right)(1+0.15A+2.91A^{2}).$$

It should be noted that the random cutting ζ can lead to a mechanical deactivation of whole regions that are still part of the model. Therefore, $\varphi_{\zeta }$ should not be interpreted as effective solid fraction $\varphi_{\mathrm{eff}}$ that represents the load bearing mass [33].

In view of the number of parameters that may play a role, we limited the structural variation to the randomization A and the cut fraction ζ and kept all other structural parameters within each data set constant (ligament aspect ratio $r_{end}/l$, ligament shape $r_{sym}^{*}$). Two data sets for ligament shapes $G_{21}$ and $G_{33}$ (see Table 1) were created, covering a large range from very low ($\varphi_{0}\sim 12\%$) to very high ($\varphi_{0}\sim 36\%$) solid fractions. Because the porosity was computed from $1-\varphi_{0}$, the porosity ranged from ~64% to ~88%.

**Figure 2:**
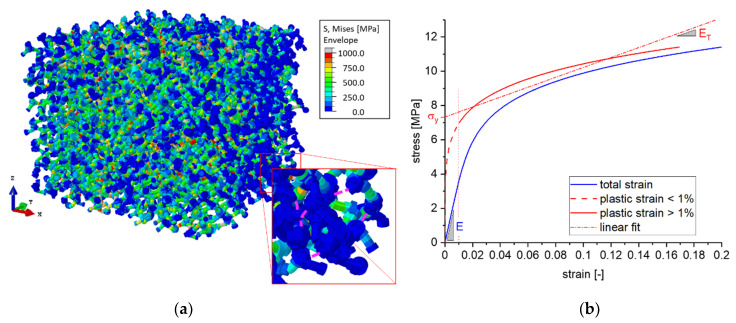
(**a**) RVE consisting of 8 × 8 × 8 unit cells with A=18% and ζ=26% after compression with 20% strain in the negative z-direction. The purple dashed curves in the magnified image shown on the right side indicate the axis of some cut ligaments. Due to the missing load transmission, the remaining dangling parts show zero stress (blue color). (**b**) Simulation output in the form of stress–strain and stress–plastic strain curves from which the macroscopic yield stress and work hardening rate are determined.

We used nanoporous gold (np-Au) as model material, because in terms of microstructure and mechanical properties this is the best investigated material of a variety of nanoporous metals reported in the literature. The chosen material behavior is plasticity with linear isotropic hardening [23]. This adds two material parameters denoted as yield stress $\sigma_{y,s}$ and work hardening rate $E_{T,s}$; the subscript s denotes that both parameters are a property of the solid phase, which makes up the 3D network. Both depend on the ligament diameter, which can be manipulated during the sample preparation of the material via the Au/Ag ratio, dealloying conditions, and heat treatment, as demonstrated in [5,32]. The elastic constants for gold are known and were kept constant for all simulations: Young’s modulus $E_{s}=80$ GPa, Poisson’s ratio $\nu_{s}=0.42$. An example of a deformed RVE (A=18%, ζ=26%) is shown in Figure 2a. The stress is evenly distributed over the length of the RVE, which indicates that the overall deformation is homogeneous despite the local structural variations due to the randomization of the ligament network.

This model makes up a set of variable inputs consisting of 5 independent parameters:

(4) $$X=\left(\varphi_{0},A,\;\zeta ,\sigma_{y,s},E_{T,s}\right).$$

For each initial solid fraction, the remaining parameters are randomly set for each simulation within the ranges 0≤A≤0.3, 0≤ζ≤0.3, $20\;\mathrm{MPa}\leq \sigma_{y,s}\leq 1000\;\mathrm{MPa}$, and $1\;\mathrm{GPa}\leq E_{T,s}\leq 10\;\mathrm{GPa}$, which cover the known range of experimental data. The random distribution of the parameters is uniform for A, ζ, $\log\;\sigma_{y,s}$, and $\log\;E_{T,s}$. Each parameter set is stored together with the job number, which uniquely connects microscopic to macroscopic compression as well as nanoindentation properties in the data processing in Section 3 and Section 4. The random choice of the parameter sets has the advantage that the parameter space is evenly filled while no parameter is computed more than once. This avoids patterns that might be unwantedly recognized by the machine learning algorithms. Furthermore, the parameter space can continued to be filled if it turns out that the number of patterns is not sufficient for the analysis. This is particularly useful when the simulations are computationally expensive. For an example where this strategy is applied in combination with artificial neural networks for solving a complex inverse problem in nanoindentation, the reader is referred to [34].

The resulting compression behavior of each pattern is represented by 5 dependent properties:

(5) $$Y=\left(E,\nu ,\nu_{p},\sigma_{y},E_{T}\right),$$

where $E,\nu ,\sigma_{y}$, and $E_{T}$ denote the macroscopic Young’s modulus, elastic Poisson’s ratio, yield stress, and work hardening rate, respectively. The computation of the plastic Poisson’s ratio $\nu_{p}$ follows [32]

(6) $$\nu_{p}=-\frac{\delta \varepsilon_{⊥}}{\delta \varepsilon_{∥}},$$

where $\delta \varepsilon_{⊥}$ and $\delta \varepsilon_{∥}$ are increments of true strain normal and parallel to the loading direction, respectively. Because $\nu_{p}$ changes during plastic compression, it is measured at 10% plastic compression strain. As demonstrated in Figure 2b, the predicted stress–plastic strain data is linearly fitted for plastic strains >1% for obtaining the macroscopic yield stress $\sigma_{y}$ and work hardening rate $E_{T}$.

### 2.2. Nanoindentation

**Figure 3:**
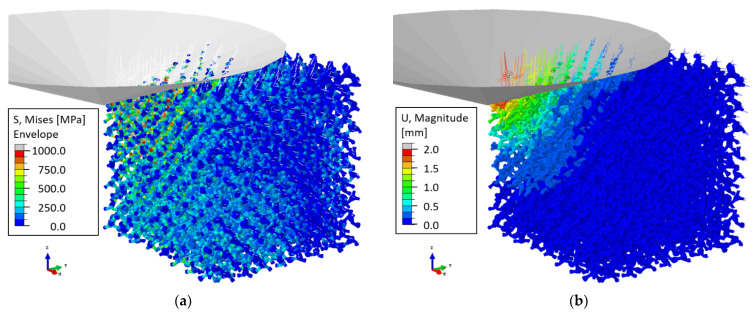
Nanoindentation model with a conical indenter displaced by 2 unit cells at a speed of 20 mm/s. Dashpots are attached at FE nodes located at free boundaries to stabilize oscillations in the dynamic simulation. Images show the deformation of the RVE at the end of the loading phase, where the color corresponds to (**a**) von Mises stress, and (**b**) displacement magnitude.

For the simulation of nanoindentation, the model described in Section 2.1 is extended by adding a conical indenter with an angle of 140.6°. For this angle, the volume-to-depth ratio of the conical indenter corresponds to that of a Berkovich tip. Details on the simulation of nanoindentation for solids and thin films can be found, e.g., in [34]. Due to the numerous ligaments that get in contact during the indentation process, an explicit dynamic analysis was required for achieving convergence. A robust load signal was produced by attaching dashpots at the free boundaries (see Figure 3) to damp elastic waves induced by the multiple contact events during the dynamic indentation process.

It can be seen from Figure 3 that the contact of the indenter, modeled as a rigid body, is established with the axis of the beam elements. Therefore, the upper half of the ligaments in contact peek out on the upper side of the indenter surface. Contact among the ligaments is not considered. In principle, this is possible in Abaqus Explicit, but the contact is limited to a pair of a rendered element surface and the axis of a second element. Preliminary studies with this indentation model revealed that such events happen rarely and at a very late stage of the indentation and, therefore, can be neglected in the total force on the indenter. It should be noted that this situation can change once we work with real microstructures and with a contact formulation that accounts for the surface of both contacting ligaments.

**Figure 4:**
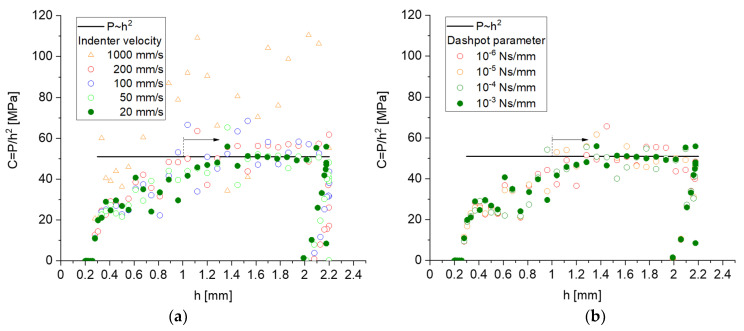
Parametric study for adjustment of (**a**) indenter velocity with smooth step function for a dashpot parameter of $10^{-3}$ Ns/mm and (**b**) dashpot parameter at fixed indenter velocity of 20 mm/s.

The calibration of the indenter velocity and the dashpot parameter is presented in Figure 4 for ligament geometry $G_{21}$ ($\varphi_{0}=0.12$) with $\sigma_{y,s}=200$ MPa, $E_{T,s}=6$ GPa, and a randomization A=0.23 [32]. For simplicity, effects of the surface energy are not included, and the cut fraction is set to ζ=0. For uniaxial compression, the predicted stress–strain curve yields the following macroscopic mechanical properties: E=1.9 GPa, ν=0.178, $\sigma_{y}=17.8$ MPa, and $E_{T}=108$ MPa.

For a conical or pyramidal indenter, the load P always increases with the square of the indentation depth h [34], as soon as the indented material acts like a continuum. Therefore, a plot of $P/h^{2}$ vs. h should tend towards a constant value. This is reached for h > 1 mm (~1 unit cell), indicating that at this depth value, a sufficient number of ligaments are in contact, and the solution homogenizes over enough microstructural elements. It can be seen from Figure 4 that the results show large scatter for high loading rates and for a low dashpot constant. The load–depth curves converged into a sufficiently steady state solution for an indenter velocity of 20 mm/s, combined with a smooth step function and a dashpot parameter of $10^{-3}$ Ns/mm. These settings were used for all following simulations, including those shown in Figure 3. The displacement magnitude in Figure 3b shows negligible deformation at the free boundaries, which suggests that the RVE is sufficiently large. With this model and parameter setting, the total CPU time per simulation is ~60 CPUh. Generating a data set with 100 simulations for a selected ligament shape requires ~1 week in real time by parallel computing on 16 CPUs.

For h>1 mm, $P_{i}/h_{i}^{2}$ values are averaged to compute the leading constant C describing the loading curve $P=Ch^{2}$. A robust hardness value can be computed by $H=P_{t}/A_{c}$, where $P_{t}=Ch_{t}^{2}$ is the load at maximum indentation depth, $A_{c}=\pi a_{c}^{2}$ is the contact area, and $a_{c}$ is the contact radius at this depth. For the example shown in Figure 3 and Figure 4, we obtain $a_{c}=5.3$ mm and a hardness of H=2.79 MPa. Thus, the hardness value is significantly lower than the macroscopic yield stress, which is $\sigma_{y}=17.8$ MPa, whereas the common assumption for foams is that $H=\sigma_{y}$ [35,36,37,38]. This motivates a detailed investigation of the dependence of $H/\sigma_{y}$ with respect to possible effects caused by the network geometry (randomness, connectivity) and elastic–plastic material properties of the ligaments, which is presented in Section 4. The data generation for the nanoindentation simulations uses the same parameter sets as those used for the simulation of macroscopic compression in Section 3, i.e., for each solid fraction, we performed 100 simulations for macroscopic compression and another 100 simulations for nanoindentation. In a few cases, the simulations of the macroscopic compression did not converge. These parameter sets were removed from both databases to avoid confusion in the analysis that combines macroscopic compression with nanoindentation data.

## 3. Macroscopic Compression

In the following sections, we reduced the dimensionality of the problem to extract relationships from our data that can be visualized, discussed, and, in the best case, modeled with simple mathematical functions. Our strategy consisted of three steps: (i) dimensional analysis, (ii) principal component analysis, and (iii) visualization and modeling of the relationship with a minimum number of inputs. The dimensional analysis [39] makes use of the physics background and the Buckingham π theorem to reduce the problem without loss of accuracy. This turned out to be a useful approach that should always be placed as a first step of feature engineering, because it ensures that the basic physics is incorporated in the input and output data, while at the same time the machine learning algorithms are relieved and their generalization capability is substantially increased [34,40,41].

Principal component analysis (PCA) was applied in conjunction with a multi-layer perceptron (MLP) algorithm using the scikit-learn package [42]. The MLP, also known as artificial neural networks, allows for the analysis of patterns consisting of multiple inputs and outputs with respect to underlying nonlinear dependencies. For details and applications, the reader is referred to [43,44,45]. After the dimensionality of the problem was reduced, comparably compact MLPs consisting of two hidden layers with 3 and 2 neurons were used for approximation and visualization of the data. This is possible, when the relationship of interest is sufficiently represented by the selected inputs.

### 3.1. Dimensional Analysis

The mechanical behavior of the RVE can written in form of dependencies for the elastic and plastic macroscopic properties

(7) $$\left(E,\nu \right)=f_{e}\left(\frac{r_{mid}}{r_{end}},\frac{r_{end}}{l},\;E_{s},\;\nu_{s},A,\zeta \right)$$

and

(8) $$\left(\sigma_{y},E_{T},\nu_{p}\right)=f_{p}\left(\frac{r_{mid}}{r_{end}},\frac{r_{end}}{l},\;E_{s},\;\nu_{s},\sigma_{y,s},\;E_{T,s},A,\zeta \right),$$

respectively. Assuming that the ligament shape is sufficiently represented by the initial solid fraction, Equations (7) and (8) simplify to

(9) $$\left(E,\nu \right)=g_{e}\left(\varphi_{0},E_{s},\;\nu_{s},A,\zeta \right),$$

(10) $$\left(\sigma_{y},E_{T},\nu_{p}\right)=g_{p}\left(\varphi_{0},\;E_{s},\;\nu_{s},\sigma_{y,s},\;E_{T,s},A,\zeta \right).$$

First, we used a priori knowledge in form of the Gibson–Ashby scaling law $E/E_{s}=C_{E}\varphi^{2}$[35]. The leading constant $C_{E}$ depends on the unit cell geometry, which in our case was defined by the diamond structure and the chosen ligament shape. To simplify Equation (9) with respect to the Young’s modulus, we can assume that the Poisson’s ratio of the ligaments has no effect on the macroscopic deformation of the RVE, which results mainly from bending of the ligaments [23]. Combining both aspects and include Equation (3) for computing the solid fraction, we can reduce Equation (9) to a dependence of only two microstructural descriptors,

(11) $$\frac{E}{E_{s}\varphi^{2}}=g_{E}^{*}\left(A,\zeta \right),$$

which can be evaluated easily by visualization of the data in a 3D plot. If such a plot confirms Equation (11), the varying ligament shape is sufficiently represented in the solid fraction φ. Furthermore, $g_{E}^{*}$ represents a generalized Gibson–Ashby law that considers the dependence from the degree of randomization and cuts of the 3D network, which is not captured simply by the solid fraction. It also extends the master curve proposed in [28], which was produced using perfectly ordered RVEs, a single solid fraction, and constant material behavior.

Along the same line of thinking, it follows for the simplification of Equation (9) with respect to Poisson’s ratio that a dimensionless macroscopic property can only depend on dimensionless microscopic quantities, i.e., the Young’s modulus $E_{s}$ plays no role. In the same way as before, we can remove a dependence of $\nu_{s}$. The macroscopic Poission’s ratio can be understood as the result of the translation of the vertical compression deformation into a lateral expansion by the architecture of the deforming 3D network, defined by A and ζ. This argument is in line with Gibson and Ashby, who stated that the Poisson’s ratio is expected to be independent of the relative density [46]. Thus, we get

(12) $$\nu =g_{\nu }^{*}\left(A,\zeta \right).$$

Concerning the increased number of independent parameters in Equation (10), dimensionality reduction would support both their understanding and modeling of their relationships responsible for the plastic response. Before going into the analysis of the data, it is useful to rewrite this equation in dimensionless form. Again, we can assume that $E_{s}$ and $\nu_{s}$ have no effect. For plasticity, this can only be assumed as long as $\sigma_{y,s}\ll E_{s}$ and $E_{T,s}\ll E_{s}$. Otherwise, we would combine comparable contributions of elastic and plastic deformation in the macroscopic response of the RVE, which requires the consideration of two dimensionless parameters for describing the elastic plastic behavior, namely $\sigma_{y,s}/E_{s}$ and $E_{T,s}/E_{s}$. Using the Buckingham π theorem [39], we can eliminate one more argument without loss of generality. One way is to normalize the macroscopic properties on the left side by their respective solid properties in the form

(13) $$\left(\frac{\sigma_{y}}{\sigma_{y,s}},\;\frac{E_{T}}{E_{T,s}},\nu_{p}\right)={\hat{g}}_{p}\left(\varphi ,\;\frac{E_{T,s}}{\sigma_{y,s}},A,\zeta \right).$$

Again, we can incorporate the Gibson–Ashby scaling law for the yield stress $\sigma_{y}/\sigma_{y,s}=C_{\sigma_{y}}\varphi^{3/2}$ [35], which yields for the first output

(14) $$\frac{\sigma_{y}}{\sigma_{y,s}\varphi^{3/2}}={\hat{g}}_{\sigma_{y}}^{*}\left(\frac{E_{T,s}}{\sigma_{y,s}},A,\zeta \right).$$

Concerning the second output of Equation (13), it is unknown which scaling is appropriate, because the work hardening rate is a slope in the stress–plastic strain diagram. Intuitively, one would follow Equation (14) in favor of an exponent of 3/2. We can answer this question together with PCA and keep the exponent β in the scaling flexible, such that

(15) $$\frac{E_{T}}{E_{T,s}\varphi^{\beta }}={\hat{g}}_{E_{T}}^{*}\left(\frac{E_{T,s}}{\sigma_{y,s}},A,\zeta \right).$$

Alternatively to Equation (13), only dependent variables are used for normalization of the output

(16) $$\left(\frac{E_{T}}{\sigma_{y}},\nu_{p}\right)={\tilde{g}}_{p}\left(\varphi ,\;\frac{E_{T,s}}{\sigma_{y,s}},A,\zeta \right).$$

The choice between the two methods of normalization depends on the potential application. Equation (16) has the advantage that all quantities on the left side are experimentally accessible, such that it could be possible to invert ${\tilde{g}}_{p}$ and to obtain some insight into material or structural properties of the nanoporous metal based on macroscopic compression testing.

### 3.2. Principal Component Analysis

At first glance, principal component analysis (PCA) [47,48] appears to be meaningless for our case, because there are no linear dependencies among the inputs that could be easily eliminated. PCA of linearly independent inputs simply translates the original inputs into a smaller number of components by linear combination. In case that each original input carries important information, this leads to a loss of information and to an increase in the predicted error in a subsequent MLP regression. In contrast, a successful reduction to a fewer number of components without a substantial increase in the prediction uncertainty shows that there is a potential for the reduction of the dimensionality of the problem and, furthermore, it delivers a feeling for the number of inputs that can be removed. The advantage of PCA is that the data can be quickly analyzed, and it becomes clear which elements of a relationship are the promising candidates for a deeper analysis.

**Figure 5:**
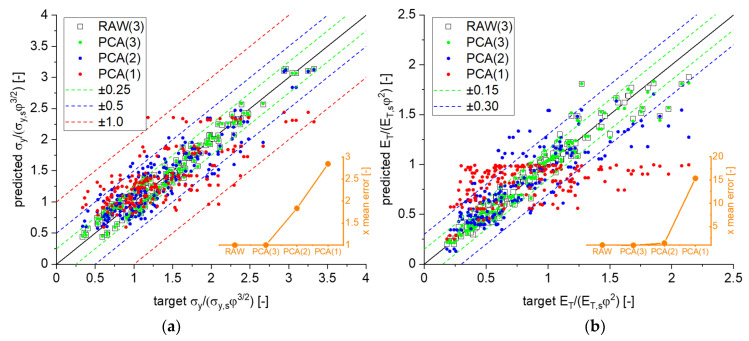
Result of PCA followed by MLP regression with a single hidden layer of 10 neurons for a decreasing number of components: (**a**) scaled yield stress, Equation (14); (**b**) scaled work hardening rate, Equation (15).

Because the equations for elasticity can be easily visualized, Equations (11) and (12) are omitted here. The mapping of PCA with an MLP regression of Equation (14) is shown in Figure 5a. For these regressions, consistently 10 neurons in a single hidden layer were used. The results for 3 components corresponded to the dimensionality of the raw input data and reproduced the accuracy of the MLP prediction without PCA, validating that no information was lost by the transformation. With reduction of the components, computed mean values of the absolute prediction error were 0.121, 0.221, and 0.342 for 3, 2, and 1 components, respectively. As can be seen from the inserted plot (orange), the error doubled with each component that was reduced. The scatter plot in Figure 5a suggests to visualize the data in form of a 3D plot, where a parametrization with one of the three inputs is required, which is presented in Section 3.4.

A first investigation of Equation (15) with 3 components and an exponent β=3/2 similar to Equation (14) led to two main groups in the scatter plot (not shown), which could be combined in a narrow scatter band by changing the exponent to β=2 (black open boxes in Figure 5b. Thus, the data suggested that the work hardening rate should be scaled in the same way as the Young’s modulus. Using this exponent, we obtained mean values of the absolute error of 0.070, 0.177, and 0.317 for 3, 2, and 1 components, respectively.

**Figure 6:**
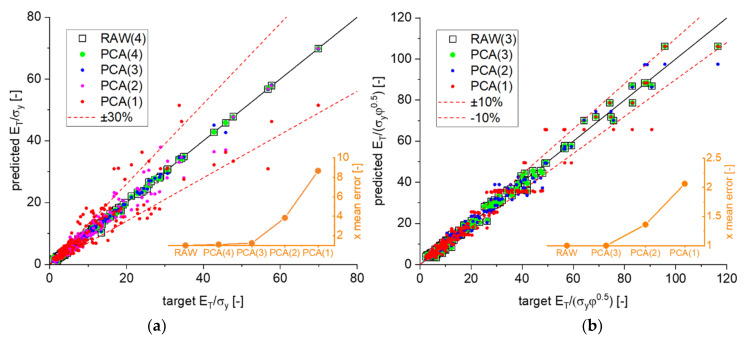
Quality of MLP regression after PCA with decreasing number of components. (**a**) $E_{T}/\sigma_{y}$, Equation (16) with ≤4 components and (**b**) $E_{T}/\left(\sigma_{y}\varphi^{1/2}\right)$ scaled according to Equation (18) with ≤3 components.

Next, it was of interest to quantify the highest possible reduction of arguments of ${\tilde{g}}_{p}$ in Equation (16). The more significant the outcome is, the better are the chances for deriving a relationship that can potentially also be solved with respect to one of the arguments. This would be a valuable aid in accessing local structural or mechanical properties from comparably simple macroscopic tests. Figure 6 presents the outcome of a PCA of ${\tilde{g}}_{p}$ followed by MLP. The PCA was first applied simultaneously to both ligament shapes $G_{21}$ and $G_{33}$. Each dimensionless parameter on the left side of Equation (16) is individually evaluated.

The importance of the correct scaling was demonstrated for the analysis of Equation (16). The results for an output in the form of the ratio $E_{T}/\sigma_{y}$, shown in Figure 6a, revealed that the argument of Equation (16) could not be easily reduced without adding considerable error. The reduction from 3 to 2 components led to a separation into two branches (reddish colors) that resulted from the two solid fractions. Further reduction did not change the result much.

However, dividing Equation (15) by Equation (14) for β=2 yields

(17) $$\frac{E_{T}\varphi^{3/2}}{\sigma_{y}\varphi^{2}}\frac{\sigma_{y,s}}{E_{T,s}}={\tilde{g}}_{p}^{*}\left(\frac{E_{T,s}}{\sigma_{y,s}},A,\zeta \right),$$

which can be rewritten as

(18) $$\frac{E_{T}}{\sigma_{y}\varphi^{1/2}}={\hat{g}}_{p}^{*}\left(\frac{E_{T,s}}{\sigma_{y,s}},A,\zeta \right).$$

For this type of scaling, shown in Figure 6b, PCA delivered almost a perfect match, independent of the number of components, suggesting that the argument of Equation (18) can be reduced to a single component. Because the output of Equation (18) can be expected to mainly depend on the corresponding ratio $E_{T,s}/\sigma_{y,s}$, the visualization can right away move to a 2D scatter plot of $E_{T}/(\sigma_{y}\varphi^{1/2})$ versus this quantity.

In contrast to the output $E_{T}/\sigma_{y}$, the plastic Poisson’s ratio $\nu_{p}$ showed a large scatter that is almost invariant to the number of components (not shown). Therefore, no further reduction of the dimensionality is possible for this parameter. This is further discussed along with the visualization of the data in Section 3.4.

### 3.3. Macroscopic Elastic Properties

**Figure 7:**
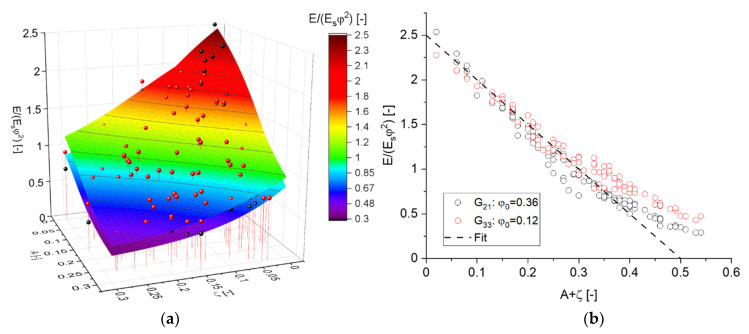
Approximation of the simulation results for the scaled Young’s modulus$\;E/(E_{s}\varphi^{2})$. Black and red spheres represent data from ligament shapes $G_{21}$ and $G_{33}$, respectively, and MLP regressions are shown as contour plot as function of randomization A and cut fraction ζ: (**a**) 3D plot of Equation (11), (**b**) quality of approximation by Equation (19).

**Figure 8:**
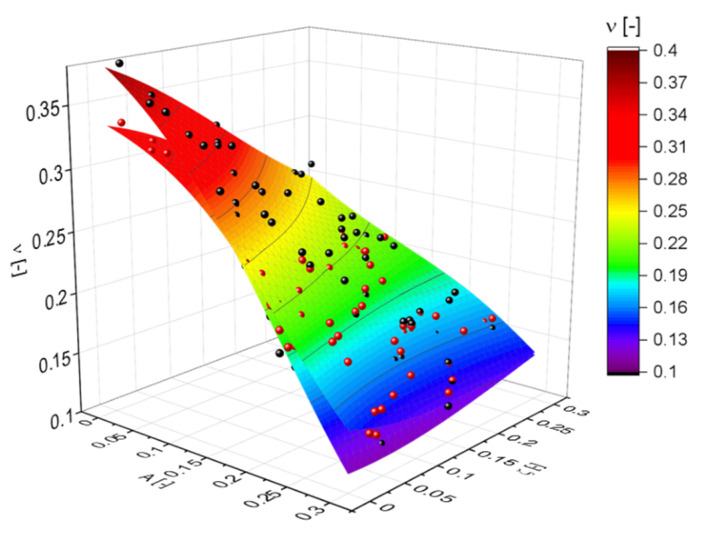
Visualization of the simulation results for the macroscopic Poisson’s ratio. Black and red spheres represent data for ligament shapes $G_{21}$ and $G_{33}$, respectively. MLP regressions of both data sets are shown as contour plots.

The dependencies for the elastic properties according to Equations (11) and (12) are visualized in Figure 7a and Figure 8. In these Figures, the randomly distributed simulation data are shown as spheres, and the predictions of the MLP regressions are shown as 3D contour plots. As can be seen from Figure 7, the scaling of Young’s modulus removes most of the effect stemming from the solid fraction, such that $g_{E}^{*}$ can be written as dependence of only two structural parameters A and ζ. The surfaces approximating the individual solid fractions are slightly shifted in the lower regions and intersect at $E/(E_{s}\varphi^{2})\sim 1.5$.

Overall, the contour plot in Figure 7a confirms the existing understanding about the effect of A and ζ, which both lower the macroscopic Young’s modulus of the ligament network [28]. Additionally, with ζ approaching the percolation threshold, one observes a smooth transition into a horizontal tangent with the *x*-axis. Interestingly, after considering both parameters in the computation of the solid fraction, the remaining effect is almost identical, as can be seen from the horizontal isolines in Figure 7a. Combining them in the *x*-axis in Figure 7b reveals where the two data sets start to separate. The dashed line indicates that up to a value of A+ζ=0.3 the data can be fitted by

(19) $$\frac{E}{E_{s}\varphi^{2}}\approx 2.5-5\left(A+\zeta \right).$$

The macroscopic Poisson’s ratio shown in Figure 8 behaves differently. Again, the effect of the randomization A is at least as strong as the effect of the cut fraction ζ. However, in agreement to the findings in [28], the cut fraction of ζ has no effect for A~0.25, while it is large for lower values of A and of opposite sign for A=0.3. The Poisson’s ratio is only slightly sensitive to φ in the regime of low values of ζ, i.e., for fully connected networks. In summary, as suggested by the PCA, the visualization in Figure 8 confirms that the dimensionality of this relationship cannot be further reduced.

### 3.4. Macroscopic Plastic Properties

**Figure 9:**
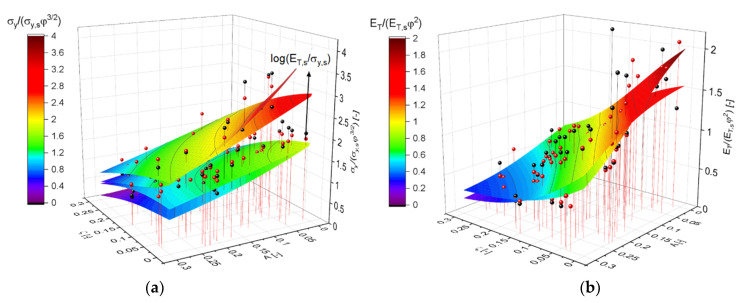
Visualization using MLP regression (shown as contour plot) along with black and red spheres corresponding to the data from ligament shapes $G_{21}$ and $G_{33}$, respectively. (**a**) Scaled yield stress after Equation (14). Each pair of contour plots represents the two solid fractions. The parametrization of $\log(E_{T,s}/\sigma_{y,s})$ corresponds to the values 0.5 and 2.5. (**b**) Scaled work hardening rate after Equation (15) with an exponent β=2, where the inputs for the MLP regression are reduced to A and ζ. The pair of planes represent the two solid fractions.

With the outcome of the PCA in mind, Equation (14) is visualized in Figure 9a. Each pair of contour plots correspond to the two solid fractions, the effect of which is captured by the scaling with $\varphi^{3/2}$. In addition to uncertainties and numerical errors, the remaining gap within each pair could be a result, e.g., of torsion that scales with φ and can have some 10% contribution to the deformation as soon as the ligaments are randomized [25]. The effect of $\log(E_{T,s}/\sigma_{y,s})$ is remarkable in all regions of the plot and is around a factor of 2, but also the dependencies of A and ζ are significant. This explains why all three parameters need to be kept for a good representation of Equation (14), as indicated by the PCA. The proper scaling of the work hardening rate with an exponent of β=2 is confirmed with Figure 9b, which is in appearance and range of values very close to that of the Young’s modulus shown in Figure 7a.

**Figure 10:**
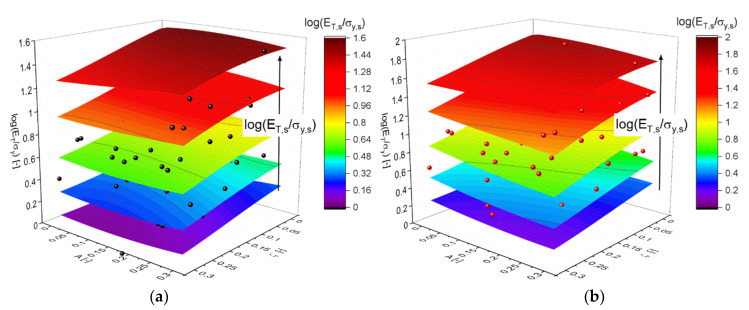
Approximation of the simulation results for the macroscopic plastic properties (spheres) by MLP regression (shown as contour plot) as functions of randomization A and cut fraction ζ: (**a**) $G_{21}$: φ=0.12, (**b**) $G_{33}$: φ=0.35.

**Figure 11:**
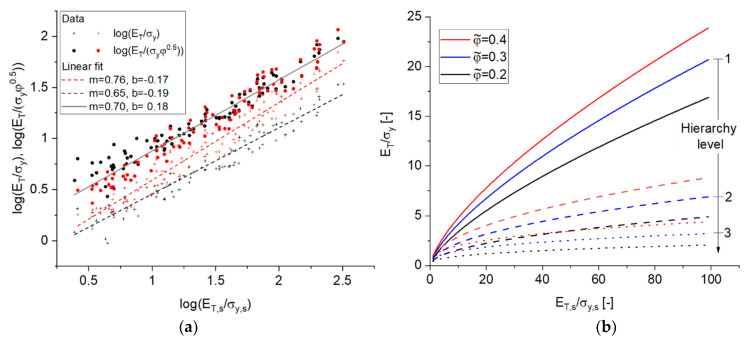
(**a**) Scaled work hardening rate to yield stress reduced to 2D plots for $E_{T}/\sigma_{y}$—Equation (16) and $E_{T}/(\sigma_{y}\varphi^{1/2})$ —Equation (18) for the two data sets $G_{21}$ (black), $\varphi_{0}=0.12$ and $G_{33}$ (red), $\varphi_{0}=0.35$. Linear fits given as y=mx+b; (**b**) plot of $E_{T}/\sigma_{y}$ for one and two levels of hierarchy according to Equations (21)–(23), respectively, showing the decreasing influence of $E_{T,s}/\sigma_{y,s}$ with increasing hierarchy level.

For obtaining a first impression on the dependence of $E_{T}/\sigma_{y}$ according to Equation (16), the data is visualized in Figure 10. The dependence of $\log(E_{T}/\sigma_{y})$ is clearly the strongest and nicely correlated with $\log(E_{T,s}/\sigma_{y,s})$, whereas A and ζ have no or only a small effect, respectively. Therefore, the data can be plotted as $\log(E_{T}/\sigma_{y})$ versus $\log(E_{T,s}/\sigma_{y,s})$ in a 2D scatter plot. The correlation shown in Figure 11 is linear over the whole range from $0.5\;\leq \log(E_{T,s}/\sigma_{y,s})\leq 2.5$, i.e., from almost perfectly plastic to strongly work hardening materials. The slope is positive, i.e., an increase in the ratio $E_{T,s}/\sigma_{y,s}$ increases the corresponding ratio $E_{T}/\sigma_{y}$ in the macroscopic behavior, which is expected. The scatter around the linear fit is, with few exceptions, ±0.1, which corresponds to 25% in a linear scaling. This scatter results in part from the additional dependence of ζ, which is visible in a tilt of the contour plots in Figure 10.

The parameters of the linear fits for $\log(E_{T}/\sigma_{y})$ depend on the solid fraction, which could be incorporated, e.g., by linear interpolation between them, because this effect is small compared to the range of $\log(E_{T}/\sigma_{y})$. Alternatively, we can make use of the scaling according to Equation (18), which removes the effect of the solid fraction, such that the two data sets are merged in the scatter plot in Figure 11a for $\log\left(E_{T}/(\sigma_{y}\varphi^{1/2}\right))$. The correlation is again linear in the log–log plot and can be fitted with

(20) $$\log(E_{T}/(\sigma_{y}\varphi^{\frac{1}{2}}))=0.18+0.7\log(E_{T,s}/\sigma_{y,s}).$$

Equation (20) can be rewritten as

(21) $$\frac{E_{T}}{\sigma_{y}}=b\sqrt{\varphi }{\left(\frac{E_{T,s}}{\sigma_{y,s}}\right)}^{\gamma },$$

with b=1.514 and γ=0.7.

Shi et al. [6] developed scaling laws for the macroscopic Young’s modulus and yield stress (in general denoted as property P) for a hierarchically nested network of n levels of the form $P_{\mathrm{net}}=b^{n}P_{s}{\tilde{\varphi }}^{n\beta }$. This results from the recursive application of the Gibson–Ashby scaling law $P_{\mathrm{eff}}=bP_{s}\varphi^{\beta }$ under the assumption of a strong self-similarity $\varphi_{\mathrm{net}}={\tilde{\varphi }}^{n}$. Here, $P_{s}$ is the mechanical property of the solid phase, $P_{\mathrm{eff}}$ is the effective (homogenized) value, and $P_{\mathrm{net}}$ is the result of the net value of P. For the work hardening to yield stress ratio as given by Equation (21), the property itself scales with an exponent $P^{\gamma }$, such that the effective properties on the next hierarchy level are $P_{\mathrm{eff},j}=b{\tilde{\varphi }}^{\beta }P_{\mathrm{eff},j-1}^{\gamma }$ with β=0.5. Therefore, $E_{T}/\sigma_{y}$ for a material with two and three levels of hierarchy is given by

(22) $$\frac{E_{T}}{\sigma_{y}}=b^{1+\gamma }\sqrt{{\tilde{\varphi }}^{1+\gamma }}{\left(\frac{E_{T,s}}{\sigma_{y,s}}\right)}^{\gamma^{2}}$$

and

(23) $$\frac{E_{T}}{\sigma_{y}}=b^{1+\left(1+\gamma \right)\gamma }\sqrt{{\tilde{\varphi }}^{1+\left(1+\gamma \right)\gamma }}{\left(\frac{E_{T,s}}{\sigma_{y,s}}\right)}^{\gamma^{3}},$$

respectively. For two levels, the total solid fraction is $\varphi ={\tilde{\varphi }}^{2}$, which ranges from 0.119 to 0.165 [6]. In this case, $\tilde{\varphi }$ ranges from 0.345 to 0.406, which changes the leading term in Equation (22) by 14%. Because of the exponent $\gamma^{2}=0.49$, a similar effect would require a variation in the material properties $E_{T,s}/\sigma_{y,s}$ by a factor of 1.33. This trend is shown in Figure 11b, for a variation of $E_{T,s}/\sigma_{y,s}$ over two orders of magnitude. If we add a third level of hierarchy, the effect of $E_{T,s}/\sigma_{y,s}$ becomes even smaller $\left(\gamma^{3}=0.343\right)$. We can therefore speculate that $E_{T}/\sigma_{y}\to 1$ with increasing number of hierarchy levels and $E_{T}/\sigma_{y}$ reduces to a function of $\tilde{\varphi }$. Section 4 shows that $E_{T}/\sigma_{y}$ is important in the interpretation of the measured hardness.

**Figure 12:**
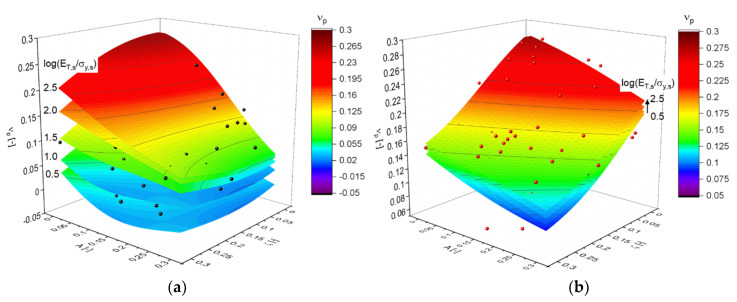
Visualization of the plastic Poisson’s ratio $\nu_{p}$ in Equation (13) for (**a**) $G_{21}$: φ=0.12 and (**b**) $G_{33}$: φ=0.35.

Finally, the dependency of the plastic Poisson’s ratio $\nu_{p}$ in Equation (13) on A, ζ, and $\log(E_{T,s}/\sigma_{y,s})$ is visualized in Figure 12. The MLP regressions shown in Figure 12a reveal that the dependence of $\nu_{p}$ on $\log(E_{T,s}/\sigma_{y,s})$ is significant for low solid fractions, while the effect of the cut fraction ζ is rather small. This changes for high solid fractions shown in Figure 12b, where the effect of $\log(E_{T,s}/\sigma_{y,s})$ is small but the effect of the cut fraction ζ has the same importance as the randomization A. In combination, both parameters can be used to tune the plastic Poisson’s ratio over a large range from ~0.3 to ~0.1. The complex dependency indicates that the multiaxial plastic deformation behavior of nanoporous metals can strongly vary and needs to be determined individually for each microstructure. Additionally, the ligament diameter and surface energy have an important effect on the plastic Poisson’s ratio, as shown in [32,49]. These experiments show a comparably large range of values from 0 to 0.2 for increasing ligament size. This range is included in the simulation data shown in Figure 12.

## 4. Nanoindentation

In this section, the dependence of the hardness was analyzed with respect to the influence of the underlying structural and mechanical properties of the nanofoam. To this end, we performed a dimensionality reduction along the same line as in Section 3 with (i) dimensional analysis, (ii) principal component analysis, and (iii) visualization and modeling of the relationship with a minimum number of inputs.

### 4.1. Dimensional Analysis

The major output of a nanoindentation experiment was the hardness H, which can be written as

(24) $$H=\bar{H}\left(r_{mid},\;r_{end},\;l,\;E_{s},\;\nu_{s},\;\sigma_{y,s},\;E_{T,s},\;A,\zeta \right).\;$$

As in Section 3.1, we represented the ligament shape defined by $r_{mid},\;r_{end},\;$ and l by the solid fraction φ and, furthermore, assumed that the hardness is governed by plastic and structural parameters, while the effect of the elastic material parameters of the comparably soft solid phase can be neglected, i.e., $\sigma_{y,s}\ll E_{s}$ and $E_{T,s}\ll E_{s}$. This reduces Equation (24) to

(25) $$H=\hat{H}\left(\varphi ,\;\sigma_{y,s},\;E_{T,s},\;A,\zeta \right).\;$$

The hardness mainly scales with the macroscopic yield stress, as this is the case for bulk materials [50]. Hence, writing Equation (25) in dimensionless form and considering that $E_{T,s}/\sigma_{y,s}$ can be replaced by a dependence of $E_{T}/\sigma_{y}$ and φ using Equation (21), this leads to a relationship that includes only macroscopic properties and structural parameters:

(26) $$\frac{H}{\sigma_{y}}={\hat{H}}^{*}\left(\varphi ,\;\frac{E_{T}}{\sigma_{y}},\;A,\zeta \right).\;$$

### 4.2. Principal Component Analysis

**Figure 13:**
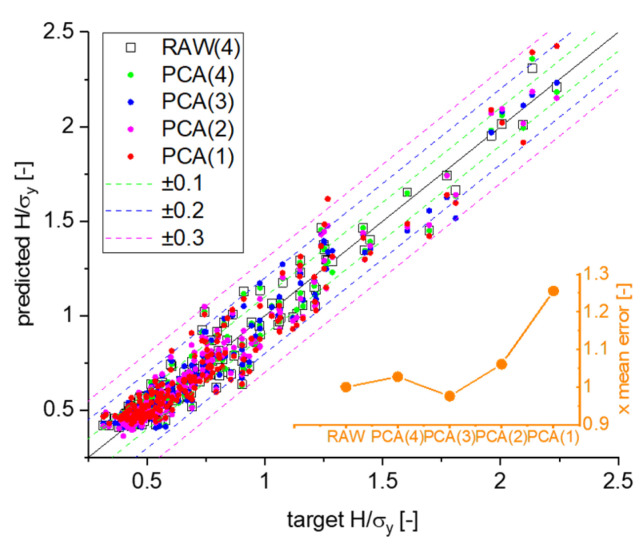
Principal component analysis of the dependence of $H/\sigma_{y}$ with respect to structural and macroscopic mechanical properties following Equation (26), combining data sets $G_{21}$ and $G_{33}$.

For further reduction of Equation (26), we used the hardness results from the indentation simulations described in Section 2.2 that were carried out with the same parameter sets as the simulations for uniaxial compression in Section 2.1. A PCA of Equation (26), shown in Figure 13, suggested that the four arguments could be reduced to one, when an uncertainty in the predicted $H/\sigma_{y}$ from ±0.1 to ±0.3 is acceptable. A reduction of the uncertainty to ±0.2 would already require at least three components. This potential for simplification is also reflected in the factor by which the absolute mean error is increased due to the reduction of the number of components, shown in the insert (orange) in Figure 13. By a reduction to a single component, this error measure is only increased by a factor of 1.3, which is a very low value compared to the results in Section 3.2.

### 4.3. Hardness

**Figure 14:**
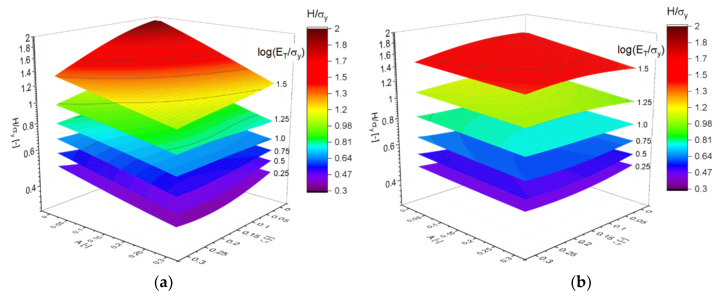

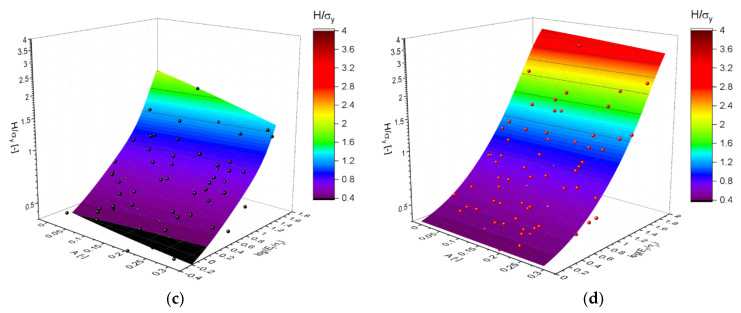
(**a**,**b**) Dependence of the normalized hardness $H/\sigma_{y}$ as function of structural properties (A,ζ ) and macroscopic material properties $\log\left(E_{T}/\sigma_{y}\right)$; (**c**,**d**) reduction of dimensionality by elimination of the cut fraction ζ, confirming that the simulation data can be represented by a simple dependence $H/\sigma_{y}\left(E_{T}/\sigma_{y}\right)$. Ligament geometries are (**a**,**c**) $G_{21}$, φ=0.12; (**b**,**d**) $G_{33}$, φ=0.35.

The quantitative dependence of the normalized hardness $H/\sigma_{y}$ as function of the structural parameters (A,ζ) and macroscopic material properties $\log\left(E_{T}/\sigma_{y}\right)$ is shown in Figure 14. The MLP regressions are shown as contour plots in Figure 14a,b, confirming that $\log\left(E_{T}/\sigma_{y}\right)$ is the most important parameter, followed by the randomization A, which has a moderate effect, whereas the effect of the cut fraction ζ can be neglected. In Figure 14c,d, the axis of the cut fraction ζ is replaced by $\log\left(E_{T}/\sigma_{y}\right)$. A small effect of the randomization A with a negative slope in the low solid fraction data can be expressed by $H/\sigma_{y}\approx {H/\sigma_{y}|}_{A=0}-0.16A$. For A=0.3, this effect is $\Delta H/\sigma_{y}\leq 0.05$, which corresponds to the uncertainty when structural effects are not taken into account. For solid fractions of typical samples with φ≈0.32, the effect caused by structural disorder is negligible.

**Figure 15:**
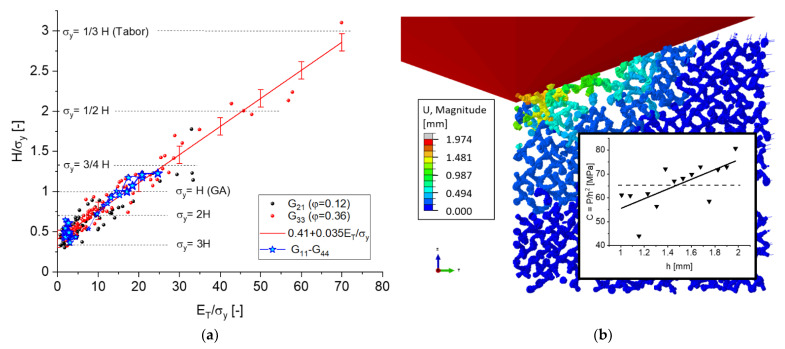
(**a**) Correlation of $H/\sigma_{y}$ with $E_{T}/\sigma_{y}$ for ligament shapes $G_{21}$ ($\varphi_{0}=0.12$ ) and $G_{33}$ ($\varphi_{0}=0.36)$. (**b**) Deformation shown for a slice of one unit cell of the indented RVE with A=0.18 and ζ=0.26, for which the stress–strain curve is shown in Figure 2b.

We could further reduce the relationship to a 2D scatter plot, shown in Figure 15, which is fitted with a linear relation

(27) $$\frac{H}{\sigma_{y}}=H_{0}^{*}+m_{H}\frac{E_{T}}{\sigma_{y}},$$

where for our data, we obtained $H_{0}^{*}=0.41$ and $m_{H}=0.035$. In this Figure, the error bars correspond to the standard deviation of ±0.11. Both ligament geometries are combined in this plot, which indicates that the dependence Equation (27) is applicable for a broad range of structures and is insensitive to microstructural parameters.

For confirmation, additional simulations for all 16 ligament geometries $G_{11}$ to $G_{44}$ [11,27] were added. These data points, entered as star symbols, represent the combinations of $r_{end}/l\in \left\{0.231,0.289,0.346,0.404\right\}$ and $r_{mid}/r_{end}\;\in \left\{0.5,0.75,1.0,1.25\right\}$ for two values $E_{T,s}/\sigma_{y,s}\;\in \left\{3.16,50.0\right\}$. This adds 32 simulations that provide an insight into possible dependencies of the ligament shape and solid fraction. The results are added in Figure 15 as blue stars, where the blue curves connect simulation results of constant $r_{end}/l$ and the line thickness increases with the value of $r_{end}/l$. All results are within the scatter of the random simulations for geometries $G_{21}$ and $G_{33}$, confirming that Equation (27) holds for all ligament shapes and solid fractions within the given scatter band. While for $E_{T,s}/\sigma_{y,s}=3.16$ the data scatter around a spot in the lower left area of the plot, the results for $E_{T,s}/\sigma_{y,s}=50$ show that with increasing $r_{mid}/r_{end}$ and solid fraction φ the data points systematically move towards larger ratios $H/\sigma_{y}$. The same applies to the random data, when we compare the range of values for the geometries $G_{21}$($\varphi_{0}=0.12$) and $G_{33}$ ($\varphi_{0}=0.35$) in black and red, respectively.

Despite the common assumption for foams $H/\sigma_{y}=1$, it seems reasonable that a porous material tends towards a bulk solid for a high solid fraction. However, it is difficult to understand that the hardness can fall below the macroscopic yield stress. This could be caused by the way the macroscopic stress–strain behavior has been translated into the material parameters $E_{T}$ and $\sigma_{y}$, as shown in Figure 2b. The macroscopic yield stress is read from the linear fit of the stress–strain curve for plastic strains >1%. This procedure removes initial nonlinearities that could be interpreted as microplasticity. However, microplasticity does not exist in our continuum model; therefore, the true yield stress is usually lower than that determined from the linear fit. In the example shown in Figure 2b, the measured yield stress determined from the linear hardening model was 7.3 MPa, whereas at 0.2% plastic strain, the stress reached a value of only 5 MPa. For H=3.2 MPa, the ratio $H/\sigma_{y}$ then changed from 0.43 to 0.64, if the yield stress at 0.2% plastic strain was used. This explains in part why the hardness can be lower than the yield stress.

It could be speculated that another contribution might stem from the reduction of the connectivity of the ligament network, as shown in Figure 15b. The RVE is characterized by a cut fraction of ζ=0.26, i.e., almost a third of the ligaments in the RVE are broken. This leads to large pores, which become comparable to the indentation depth and the contact radius. When a microstructural length and the indentation depth are of the same order, the simulation shows a size effect. For 3D networks, this problem becomes relevant when approaching the percolation threshold and is difficult to solve [51]. An increase in the normalized indentation load $P/h^{2}$, shown as insert in Figure 15b, apparently confirms this effect. However, one would then also expect a systematic bias in the data in the form of a dependence of the cut fraction, i.e., $H/\sigma_{y}\to 1$ for ζ→0, but such a trend is not present in Figure 14a,b. It is therefore possible that values $H/\sigma_{y}<1$ exist. Because this has important implications on the interpretation of hardness data of foams in general, an in-depth investigation should be the scope of future work.

## 5. Summary and Conclusions

Nanoporous metals with their complex microstructure represent an ideal candidate for method developments that combine data and AI. With a few parameters controlling the sample preparation, it is possible to tune the microstructure and macroscopic mechanical properties within a large design space. This includes, among others, the solid fraction, ligament size, and the connectivity density. It has been recently demonstrated that the versatile dealloying process allows hierarchically organized nanoporous metals with superior macroscopic properties to be produced compared to those with only one hierarchy level [6]. Via the microstructure, it is possible to tune the macroscopic properties, such as Young’s modulus, yield strength, elastic and plastic Poisson’s ratio, and hardness in wide ranges. This makes this class of materials not only attractive for various applications, such as sensing or actuation in combination with light weighting, but it is also an ideal science case for the demonstration of the capabilities of dimensionality reduction methods.

To this end, the generation of ~200 data sets for macroscopic compression and nanoindentation was realized with the help of an efficient FE-beam modeling technique. The parameter space consists of five independent inputs (microstructure, material parameters) and six dependent outputs (macroscopic compression behavior and hardness). It was systematically analyzed in three steps by means of a dimensional analysis including a priori knowledge about the problem at hand, principal component analysis, and visualization. In the latter two steps, machine learning served as key for analyzing the existence and quality of approximations on the presented data sets.

From the outcome, we conclude that, independent of the size of the data set, it is always recommendable to start with a dimensional analysis. This ensures that the analyzed dependency is formulated in a physically reasonable manner and it allows the dimensionality of the problem to be reduced by usually two quantities in quasi static mechanics or by three for dynamic problems. At this stage, it is advisable to incorporate *a priori* knowledge from the literature or by reasoning, which can further simplify the problem considerably. How well this has been done and by how many components the dependency can be further reduced can be easily tested by machine learning in combination with principal component analysis. If no deeper understanding is needed, the outcome in the form of a black box would already be a sufficient computer model of the relationship hidden in the presented data.

Deeper insight can be gained by visualization, which is also supported by machine learning. Here, the multilayer perceptron first approximates the design space from the randomly distributed data and then is applied for continuous mapping along selected inputs in the form of contour plots. This serves the validation of the previous steps as well as for a better understanding of the quantitative dependence of a specific output, e.g., the hardness to yield stress ratio, of inputs, e.g., the randomization or mechanical properties. In this way, the major dependences can be identified from a limited number of data and unimportant inputs can be eliminated. Furthermore, one obtains a measure for the uncertainty due to ignored inputs that have a non-negligible effect.

For the scientific case at hand, which is the microstructure–property relationship of nanoporous metals, there are several important findings, which are applicable not only to Au but to any metal, as long as it can be described with the chosen elastic–plastic material behavior and microstructure. Our analysis showed that the ratio of the work hardening rate to the yield stress $E_{T,s}/\sigma_{y,s}$ represents a key property that can mapped to the corresponding macroscopic ratio $E_{T}/\sigma_{y}$ in a log–log scaling. It is therefore possible to invert this relationship for measured macroscopic behavior, which allows one to gain an important insight in the amount of work hardening present in the solid phase, relative to its yield stress. Work hardening implies storage of defects in the nanoscaled ligaments, and its existence has been a matter of debate. The derived relationship can help to quantitatively underpin speculations that are in favor [52] or contradict [13,23] the mechanistic model of dislocation starvation in nanosized metallic objects simply by translating the macroscopic test data into those of the solid phase.

In addition to the known Gibson–Ashby scaling laws for Young’s modulus and yield strength, one for the work hardening rate is added, which uses the same exponent of 2 as the Young’s modulus. This is unexpected, because the work hardening rate is a slope defined by two flow stresses at different plastic strains and the yield stress, which is one of them, scales with an exponent of 1.5. Additionally, the appearance and range of the relationship as functions of randomization and cut fraction are very similar to that of the Young’s modulus.

Another important finding is the linear relation between $H/\sigma_{y}$ and $E_{T}/\sigma_{y}$. The common assumption that for hardness testing of foams $H=\sigma_{y}$ [35], which is also used in the interpretation of nanoindentation of np-Au [37,38], turned out to be a special case for $E_{T}/\sigma_{y}\sim 17$. The range of the $\;H/\sigma_{y}$ data is surprisingly large and exceeds the common values for porous and bulk solids of 1 and 3, respectively, towards lower values: A large number of data are within the range $0.5\leq H/\sigma_{y}\leq 1$. This can in part be explained by how the macroscopic stress–plastic strain curve is modeled. Another reason could be a size effect that results from large pores for samples with very low connectivity, but it appears that still values of $H/\sigma_{y}<1$ exist. Because this has important implications on the interpretation of hardness data, an in-depth investigation will be the scope of future work.

Finally, for hierarchic materials with a nested network [6], our results suggest that the effect of $E_{T,s}/\sigma_{y,s}$ becomes small or even negligible with respect to $E_{T}/\sigma_{y}$. With increasing levels of hierarchy, it can be expected that the normalized hardness $H/\sigma_{y}$ changes from a dependence of $E_{T}/\sigma_{y}$, which holds for a common nanoporous metal, towards a dependence of the solid fraction φ.

## Figures and Tables

**Table 1:** Ligament shapes used for the generation of two data sets in the low and a high solid fraction regime, respectively.

Shape	$r_{sym}^{*}$	$r_{end}/l$	$\varphi_{0}$
$G_{21}$	0.5	0.289	0.1232
$G_{33}$	1.0	0.346	0.3574

## Data Availability

The data sets analyzed in this work are available via the repository TORE (https://tore.tuhh.de/ accessed on 3 April 2021) published under https://doi.org/10.15480/336.3411.
